# Prognostic factors in H7N9 avian influenza: a systematic review based on case reports

**DOI:** 10.1186/s12879-026-12908-4

**Published:** 2026-02-18

**Authors:** Fengying Li, Kai Song, Xiaobin Ren

**Affiliations:** 1 Department of Clinical Laboratory, Sir Run Run Shaw Hospital, Zhejiang University School of Medicine, 3 East Qingchun Road, Hangzhou, Zhejiang Province 310016 People’s Republic of China; 2 Zhejiang Provincial Engineering Research Center for Innovative Instruments in Precise Pathogen Detection, Hangzhou, Zhejiang Province 310016 People’s Republic of China; 3 Institute of Infectious Disease Control and Prevention, Hangzhou Center for Disease Control and Prevention (Hangzhou Health Supervision Institution), 568 Mingshi Road, Hangzhou, Zhejiang Province 310021 People’s Republic of China

**Keywords:** H7N9 influenza, Mortality, Clinical characteristics, Systematic review

## Abstract

**Objective:**

The H7N9 avian influenza virus, identified in China in 2013, has posed a significant threat to public health due to its high mortality rate. This systematic review aims to evaluate the clinical characteristics and mortality risk factors of H7N9 patients.

**Methods:**

English and Chinese databases (PubMed, Web of Science, Embase, CNKI, VIP, Wanfang) were searched for studies on laboratory-confirmed H7N9 cases with available data on symptom onset, diagnosis time, clinical features, oseltamivir administration, and outcomes. Univariate and multivariate analyses were performed on the pooled case data to assess the relationship between clinical factors and mortality risk.

**Results:**

A total of 166 studies including 237 H7N9 cases were analyzed, with an overall mortality rate of 41.77%. Univariate analysis showed higher mortality in patients with advanced age ≥ 66 years (62.50%), those with underlying diseases (60.20%), those who received oseltamivir ≥ 8 days after symptom onset (54.17%), and those diagnosed ≥ 11 days after onset (62.75%), whereas patients treated with oseltamivir within 2 days of onset had the lowest mortality (17.39%). Multivariate analysis identified advanced age ≥ 66 years (OR = 3.10, 95% CI: 1.07–8.99, *P* = 0.037) and delayed oseltamivir administration after symptom onset (OR = 4.63, 95% CI: 1.12–19.18, *P* = 0.034) as independent predictors of mortality, while sex, underlying diseases, and onset-to-diagnosis time were not statistically significant.

**Conclusion:**

Older age and delayed initiation of oseltamivir are key independent predictors of mortality in H7N9 infection. Prompt diagnosis is crucial to facilitate early antiviral treatment, which may improve survival. Future prospective studies are needed to validate these findings and optimize clinical management.

**Clinical trial registration:**

Not applicable.

**Supplementary Information:**

The online version contains supplementary material available at 10.1186/s12879-026-12908-4.

## Background

Since the first outbreak of H7N9 avian influenza in China in 2013, the H7N9 virus has raised significant concerns in the global public health community [1]. The virus is primarily transmitted from poultry to humans and typically manifests as severe respiratory illnesses such as pneumonia, acute respiratory distress syndrome (ARDS), and multiple organ failure [2, 3]. The rapid spread and high mortality rate of the H7N9 virus have made it a serious threat to global public health. By April 2020, the World Health Organization (WHO) reported 1,568 laboratory-confirmed cases of H7N9, with 616 fatalities, yielding a mortality rate of 39% [4]. Given the public health relevance of H7N9 in human avian influenza and the availability of a sufficient number of case reports with analyzable prognostic information, this systematic review focused exclusively on the H7N9 subtype.

The clinical prognosis of H7N9 infection may be influenced by multiple factors, including advanced age, underlying health conditions, gender, and antiviral treatment. Generally, patients of advanced age face a higher risk of death, and underlying conditions such as hypertension, diabetes, and chronic respiratory diseases may exacerbate the course of illness. Additionally, early diagnosis and treatment are crucial for improving survival rates, with timely administration of antiviral medication, particularly oseltamivir, potentially aiding in better outcomes. Although many studies mention these factors, it remains unclear whether they truly affect patient prognosis. In particular, the interaction among these factors and their comprehensive impact on prognosis warrant further in-depth research.

By reviewing existing research, this study aims to offer insights into the clinical features and mortality risks of H7N9 patients, with a particular focus on how different clinical interventions may impact prognosis. The study seeks to provide valuable insights to help optimize treatment plans for avian influenza patients, ultimately offering support for clinical decision-making and improving patient outcomes.

## Methods

### Protocol registration and search strategy

The systematic review followed the Preferred Reporting Items for Systematic Reviews and Meta-Analyses (PRISMA) guidelines and the PRISMA flowchart was used for data organization [5](see Supplementary Table S1 online). The study protocol was registered in the International Prospective Register of Systematic Reviews (PROSPERO) under the registration number CRD4202455073.

Given that most H7N9 avian influenza cases have occurred in China, this study searched both English-language and Chinese-language databases to ensure comprehensive literature coverage. We searched three major English databases (PubMed, Web of Science, and Embase) and three Chinese databases (CNKI, Wanfang Database and VIP Information Database). To minimize the risk of missing relevant studies, we used a highly sensitive search term, “Influenza A Virus H7N9 Subtype OR H7N9 subtype OR H7N9 subtypes OR H7N9 Virus OR H7N9 Viruses OR Virus H7N9 OR H7N9 OR H7N9 avian influenza”. The search covered the period from the emergence of H7N9 through March 31, 2024, including various study types. In addition, the reference lists of all eligible studies and pertinent reviews were scrutinized to identify potentially overlooked studies. Possible gray literature was also searched using Google Scholar to identify additional relevant studies. Additionally, the World Health Organization (WHO) website and regional health department websites were examined to find pertinent articles and reports. Only peer-reviewed articles were included for reliability, with two researchers independently screening and verifying data during extraction.

### Eligibility criteria

The criteria for studies to be included in the review were as follows: (1) Patients with laboratory-confirmed H7N9 avian influenza (by PCR or gene sequencing); (2) Studies reporting oseltamivir use, including whether it was administered and the timing after symptom onset, or reporting the time from symptom onset to diagnosis confirmation; (3) Studies reporting patient outcomes (survival or death). Exclusion criteria were: (1) Diagnostic methods other than PCR or genetic sequencing (e.g., studies using colloidal gold for diagnosis); (2) Studies lacking information on oseltamivir use (administration and timing) or time from symptom onset to diagnosis confirmation; (3) Studies involving co-infections with other pathogens; (4) Studies not providing clear prognosis data for cases.

### Data extraction and quality assessment

Two reviewers independently extracted data from eligible studies. The extracted data included information about the first author’s name, year of publication, country, patient age, gender, underlying medical conditions (including common conditions like hypertension, diabetes mellitus, chronic respiratory diseases, and other diseases that may affect patient outcomes), onset of illness, whether the patient used oseltamivir, time from onset to diagnosis, timing from symptom onset to oseltamivir administration, patient outcomes (survival or death). One author undertook the initial selection of studies, and another reviewed the selection. All corresponding data were manually entered into an Excel database. Any discrepancies were resolved through discussion with a third author until a consensus was reached.

The quality of the included studies was assessed using the Joanna Briggs Institute (JBI) Critical Appraisal Checklist for Case Reports. In alignment with the specific requirements of this study, items 7 and 8 were deemed not directly relevant and were excluded, reducing the checklist from 8 items to 6. Each item was scored with 1 point if fully reported, and 0.5 points were assigned for partially reported items. Studies scoring below 4 were rated as low quality, 4–5 as moderate quality, and above 5 as high quality [6].

### Data analysis

All extracted data were entered into a structured Excel spreadsheet and analyzed with SPSS version 19.0. Univariate analyses were performed to assess the association between potential prognostic factors and mortality in H7N9 patients. Specifically, the relationship between sex and the presence of underlying diseases with mortality was evaluated using the chi-square test. Age, oseltamivir administration status, time from onset to oseltamivir administration, and time from onset to diagnosis were treated as three-group variables and compared using the chi-square test, with Bonferroni correction applied to control for false-positive results due to multiple comparisons (adjusted significance level *P* = 0.0167).

For multivariate analysis, variables were included if they were statistically significant in univariate analysis or considered potentially clinically relevant. Binary logistic regression was performed to identify independent predictors of mortality, including only cases with complete information for the five predictive variables (age, sex, presence of underlying diseases, time from onset to oseltamivir administration, and time from onset to diagnosis). Model stability was assessed by calculating the events per variable (EPV) ratio, with an EPV ≥ 10 indicating an adequately powered model. Multicollinearity among predictors was examined using the variance inflation factor (VIF), and a VIF < 5 was considered acceptable.

Model fit was evaluated using the Hosmer–Lemeshow goodness-of-fit test, with *P* > 0.05 indicating adequate fit. Unless otherwise specified, all statistical tests were two-sided, with *P* < 0.05 considered statistically significant.

## Results

### Literature search

**Fig. 1:**
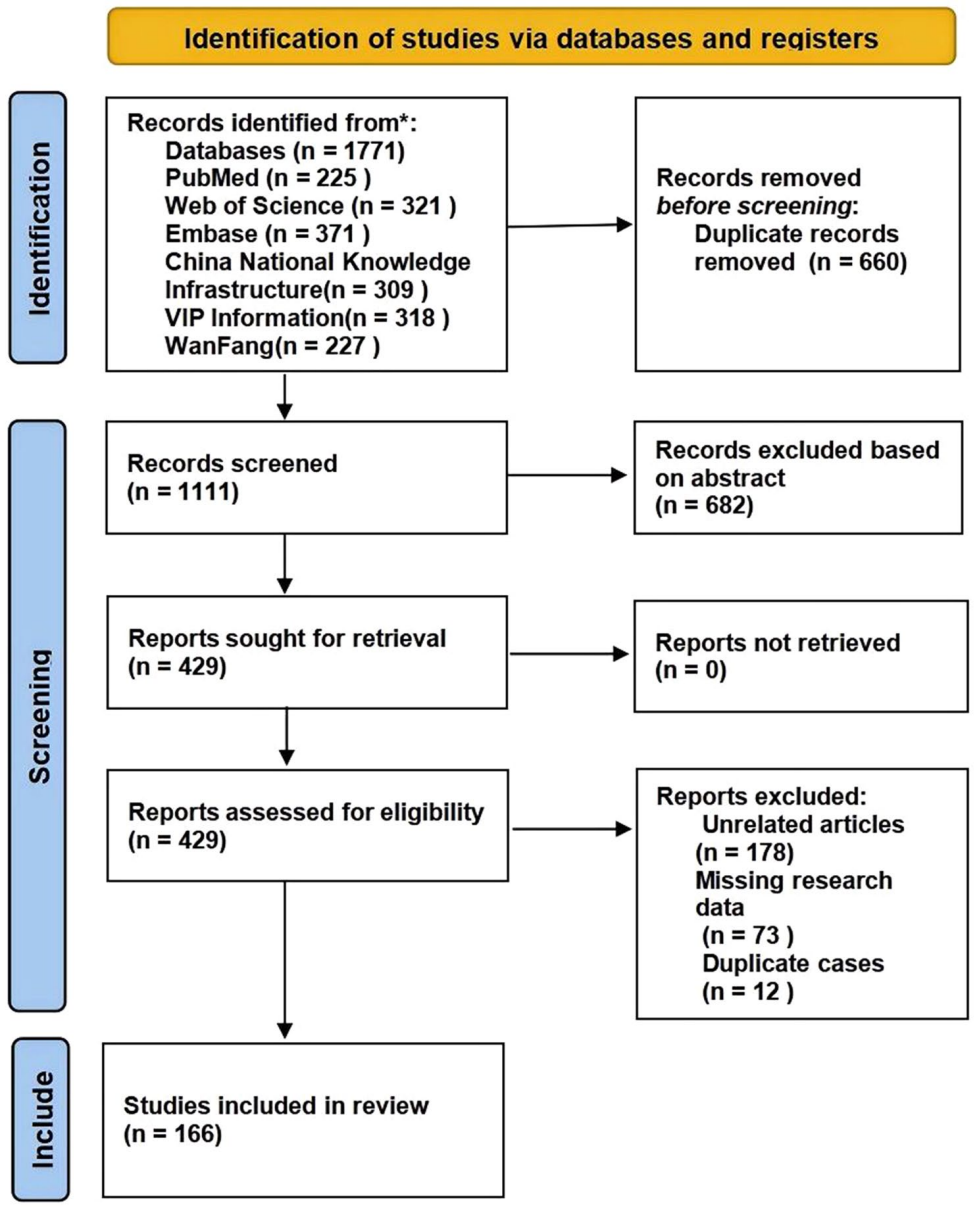
Flowchart of study identification in the systematic literature review on prognostic factors in human infection with H7N9 avian influenza

A total of 1,771 reports were initially retrieved from the databases, with 660 identified as duplicates. After removing duplicates and conducting an initial screening, 429 studies were considered potentially eligible. A thorough review further refined the selection, resulting in the inclusion of 166 articles encompassing 237 cases that met the study’s criteria. The complete search strategy for each database is detailed in Supplementary Figure S1-S6 and Table S2. The systematic review process is illustrated in the PRISMA flowchart (Fig. 1).

A total of 1,771 reports were initially retrieved from the databases, with 660 identified as duplicates. After removing duplicates and conducting an initial screening, 429 studies were considered potentially eligible. A thorough review further refined the selection, resulting in the inclusion of 166 articles encompassing 237 cases that met the study’s criteria. The complete search strategy for each database is detailed in Supplementary Figure S1-S6 and Table S2. The systematic review process is illustrated in the PRISMA flowchart (Fig. 1).

Of the included articles, 6 were descriptive studies based on case information, while the remainder were case reports. Among these, 20 were of high quality, 70 were of moderate quality, and 76 were of low quality. With the exception of one case from Malaysia and two from Hong Kong, all other cases originated from mainland China. Basic information on the included articles and cases is summarized in Table 1, while more detailed data are presented in Supplementary Table S3. References [7–169] are cited in Supplementary Table S3, which provides detailed case-level information for the included studies.

**Table 1:** Results of univariate analyses examining the associations between selected clinical characteristics and patient prognosis (survival vs. death). Categorical variables were compared using the chi-square (χ^2^) test. Sample sizes vary due to missing or incomplete data: age (n = 237), gender (n = 237), underlying diseases (n = 146), oseltamivir treatment (n = 165), onset-to-diagnosis time (n = 227)

Characteristics		*N*	Prognosis		χ² value	*P* value
			Survival	Death		
Age		237				
	≤ 18	13	11	2	15.250	*<* 0.001
	19–65	168	106	62		
	≥ 66	56	21	35		
Gender		237				
	Male	161	88	73	1.113	0.291
	Female	76	50	26		
Underlying diseases		146				
	Yes	98	29	59	7.932	0.005
	No	48	31	17		
	Yes ≤2d	23	17	4	10.597	0.005
Oseltamivir treatment		165				
	3-7d	86	58	28		
	≥ 8d	48	22	26		
	No	8	2	6	3.179	0.075
Onset-to-diagnosis Time		227				
	≤ 2d	23	18	5	9.053	0.003
	3-10d	153	94	59		
	≥ 11d	51	19	32		

## Univariate analysis

### Age, gender, underlying diseases and prognosis

A total of 166 studies with 237 cases provided information on sex, age, and prognosis. The overall case mortality rate was 41.77% (99/237). The mortality rates for H7N9 avian influenza cases were observed to be 15.38% (2/13) in individuals aged 18 years and below, 36.90% (62/168) in those aged 19 to 65 years, and 62.50% (35/56) in those of patients of advanced age (≥ 66 years). The overall difference in case mortality rates among the three age groups was statistically significant (χ²=15.250, *P <* 0.001), with the mortality rate in the group of advanced age significantly higher than in the other two groups (χ²=9.418, *P* = 0.002; χ² = 11.207, *P* = 0.001). No statistically significant difference was observed between the age groups of 18 years and below and 19 to 65 years (χ² = 2.445, *P* = 0.118).

The mortality rate was 45.34% (73/161) for male cases and 34.21% (26/76) for female cases. The difference in mortality rates between the two groups was not statistically significant (χ² = 1.113, *P =* 0.291).

A total of 103 studies with 146 cases provided information on underlying diseases and prognosis. The mortality rate for cases with underlying diseases was 60.20% (59/98), while the mortality rate for cases without underlying diseases was 35.42% (17/48). The mortality rate was higher in the group with underlying diseases, and the difference was statistically significant (χ² = 7.932, *P* = 0.005). Results are shown in Table 1.

### Oseltamivir administration and prognosis

A total of 122 studies with 165 cases provided information on oseltamivir administration and prognosis. Oseltamivir was administered to 157 cases, while 8 cases did not receive this treatment. The mortality rate for cases treated with oseltamivir for antiviral therapy was 36.94% (58/157), while the mortality rate for cases not treated with oseltamivir was 75.00% (6/8). The difference in mortality rates between the two groups was not statistically significant (χ² = 3.179, *P* = 0.075).

Further analysis showed that the mortality rate for cases treated with oseltamivir within 2 days of onset was 17.39% (4/23), the mortality rate for cases treated between 3–7 days of onset was 23.56% (28/86), and the mortality rate for cases treated 8 days or more after onset was 54.17% (26/48). The difference in mortality rates among the three groups was statistically significant (χ² = 10.597, *P* = 0.005, Table 1). The mortality rate for cases treated with oseltamivir 8 days or more after onset was higher than those treated within 2 days and between 3–7 days of onset (χ² = 8.619, *P* = 0.003; χ² = 5.979, *P* = 0.014). The mortality rates between the group treated with oseltamivir within 2 days of onset and the group treated between 3 to 7 days of onset showed no statistically significant difference (χ² = 2.013, *P =* 0.156).

### Onset-to-diagnosis time and prognosis

A total of 161 studies with 227 cases provided information on onset-to-diagnosis time and prognosis. The mortality rate for cases diagnosed within 2 days of onset was 21.74% (5/23), for those diagnosed between 3–10 days it was 38.56% (59/153), and for those diagnosed 11 days or more after onset, it was 62.75% (32/51). The difference in mortality rates between the group diagnosed 11 days or more after onset and those diagnosed within 3–10 days (χ² = 9.053, *P* = 0.003) and within 2 days (χ² = 10.662, *P* = 0.001) was statistically significant. However, no statistically significant difference was observed between the groups diagnosed within 2 days and between 3 and 10 days (χ² = 2.445, *P* = 0.118). Results are shown in Table 1.

### Multivariate analysis

A total of 90 cases with complete data for the five predictive variables (age, sex, presence of underlying diseases, onset-to-diagnosis time, and onset-to-oseltamivir administration) were included in the multivariate analysis. Among these cases, 47 deaths were reported, resulting in an EPV ratio of 9.4—slightly below the commonly recommended threshold of 10 for stable logistic regression estimates. The VIF values ranged from 1.041 to 2.118, indicating no significant multicollinearity among the variables.

**Fig. 2:**
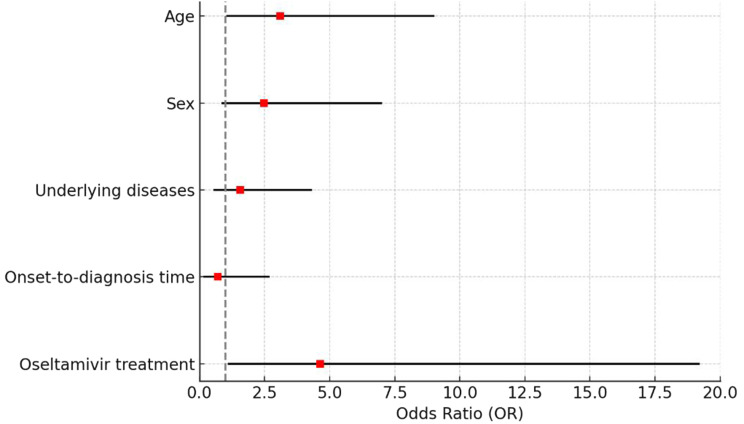
Forest plot of odds ratios from multivariate analysis for factors associated with H7N9 mortality. Odds ratios (Exp(B)) and 95% confidence intervals are shown for five variables included in a multivariate binary logistic regression model: age, sex, underlying diseases, onset-to-diagnosis time, and timing of oseltamivir administration. A total of 90 cases with complete data were included

Binary logistic regression analysis revealed that advanced age (OR = 3.10, 95% CI: 1.07–8.99, *P* = 0.037) and the time from onset to oseltamivir administration (OR = 4.63, 95% CI: 1.12–19.18, *P* = 0.034) were independently associated with mortality in H7N9 cases. In contrast, sex, presence of underlying diseases, and onset-to-diagnosis time were not significantly associated with prognosis (all *P* > 0.05). Detailed results are presented in Table 2, and a forest plot illustrating these associations is shown in Fig. 2.

**Table 2:** Results of a multivariate logistic regression analysis including age, sex, presence of underlying diseases, onset-to-diagnosis time, and timing of oseltamivir administration. A total of 90 cases with complete data for all five variables were included

Variables	B	S. E.	Sig.	Exp(B)	95.0% C.I.for EXP(B)	
					Lower	Upper
Age	1.131	0.543	0.037	3.099	1.069	8.988
Sex	0.908	0.528	0.086	2.480	0.880	6.983
Underlying diseases	0.444	0.516	0.390	1.559	0.567	4.287
Onset-to-diagnosis time	− 0.0371	0.687	0.589	0.690	0.180	2.651
Oseltamivir treatment	1.533	0.725	0.034	4.634	1.119	19.183
Constant	-4.233	1.570	0.007	0.015		

To further evaluate model fit, the Hosmer–Lemeshow goodness-of-fit test was performed. The results indicated good model fit (χ² = 6.728, df = 7, *P* = 0.458), further supporting the reliability of the regression analysis.

## Discussion

In this study, we identified advanced age and delayed antiviral therapy as the major determinants of mortality in patients with H7N9 infection. Patients of advanced age exhibited a substantially increased risk of death, and delayed initiation of antiviral therapy was significantly associated with poorer outcomes. Although onset-to-diagnosis time was not an independent predictor in the multivariate analysis, it remains clinically relevant, as timely diagnosis facilitates earlier therapeutic decision-making and the prompt initiation of antiviral treatment. Overall, our results highlight that both host vulnerability (age-related risk) and treatment timeliness, including early diagnosis, are pivotal factors influencing survival in H7N9 infection.

The findings of this study indicate that advanced age and the time from disease onset to oseltamivir administration are closely associated with mortality in patients with H7N9 infection, which is consistent with previous reports. Both this study and Zheng et al. [170]. demonstrated that advanced age have a significantly increased risk of death, highlighting the importance of advanced age as a key prognostic indicator. In the multivariate analyses of both studies, advanced age and delayed antiviral therapy were identified as independent predictors of mortality, further emphasizing the critical role of timely antiviral intervention in the management of H7N9 infection. Compared with the study by Zheng et al., our univariate analysis additionally showed that the presence of underlying diseases was significantly associated with mortality, although this association did not persist in multivariate analysis. This discrepancy may partly be attributed to differences in study populations and the heterogeneity inherent in case reports, as well as the limited ability to fully control for confounding factors across diverse clinical settings. Moreover, fundamental differences in study design may also contribute: Zheng et al. analyzed a single-center real-world cohort with complete and relatively homogeneous clinical data, allowing for clearer estimation of independent effects, whereas our study integrated case reports and case series from varied clinical environments, variations in treatment timing, healthcare resources, supportive care, disease monitoring, and reporting quality inevitably introduced heterogeneity and constrained confounder control. Despite these differences, both studies are clinically aligned in showing that advanced age and delayed antiviral therapy are associated with increased mortality in patients with H7N9 infection. This consistency reinforces the rationale for prioritizing age assessment and early antiviral intervention in clinical decision-making and further underscores the need for future large-scale, high-quality prospective studies to validate these findings.

This systematic review has several limitations. First, the majority of included studies were case reports or case series without rigorous study designs, which may introduce selection bias, as such reports often focus on severe or atypical cases. Second, heterogeneity in the reporting of demographic, clinical, and treatment information complicated data standardization. Third, because most data were derived from individual case descriptions, we were unable to fully adjust for confounding factors, and variations in healthcare settings and supportive care could not be controlled, thereby limiting causal inference. In addition, the lack of detailed survival time information in most reports precluded time-to-event analyses, restricting our modeling strategy to binary outcome regression. Moreover, although multicollinearity was not statistically evident, residual interdependence among clinically related variables cannot be entirely excluded. Additionally, most reports did not provide information on the use of antiviral agents other than oseltamivir, and missing data as well as limited sample size may constrain generalizability. Nearly all cases were from mainland China, with only three cases reported elsewhere, and viral subtype information was largely absent; therefore, geographic variability and viral mutations could not be assessed, which may limit external validity. Finally, although a comprehensive literature search was conducted, publication bias remains possible. Furthermore, quality-stratified analysis should have been performed to verify the robustness of the findings; however, the proportion of high-quality studies was low (approximately 12%). Conducting stratified analysis would have resulted in severely imbalanced sample sizes, potentially producing unstable or biased effect estimates, and was therefore not implemented. Although the EPV was acceptable, the relatively modest sample size warrants caution and highlights the need for validation in larger cohorts.

In conclusion, this study identifies advanced age and prolonged onset-to-oseltamivir time as key determinants of mortality in patients with H7N9 infection, underscoring the importance of prioritizing early antiviral intervention, particularly in elderly patients. While early diagnosis remains essential for facilitating timely clinical decision-making and enabling prompt initiation of treatment, our findings suggest that the greatest prognostic benefit may lie in rapid implementation of antiviral therapy following diagnosis. Thus, early diagnosis and early treatment should be viewed as complementary components within the same continuum of care. Future large-scale, prospective multicenter studies incorporating viral subtype and host immune profiles are warranted to validate these findings and further optimize clinical management strategies.

## Supplementary Information

Below is the link to the electronic supplementary material.

Supplementary Material 1

## Data Availability

Data is provided within the manuscript or supplementary information files.

## Abbreviations

ARDS: Acute Respiratory Distress Syndrome
WHO: World Health Organization
PRISMA: Preferred Reporting Items for Systematic Review and Meta-analysis
PROSPERO: Prospective Register of Systematic Reviews
PCR: Polymerase Chain Reaction
JBI: Joanna Briggs Institute
