# Supplementary material for: Prognostic factors in H7N9 avian influenza: a systematic review based on case reports
Source: BMC Infect Dis. 2026 Feb 18;26:631. doi: 10.1186/s12879-026-12908-4 (PMC13020402; doi:10.1186/s12879-026-12908-4)
Supplement: Supplementary file 1 — Supplementary Material 1 [file 12879_2026_12908_MOESM1_ESM.pdf]

Supplementary Figure S1：Search strategy for Pubmed database

History and Search Details Download Delete

Search	Actions	Details	Query	Results	Time
#16	•••	˅	Search: **((((((Influenza A Virus H7N9 Subtype[MeSH Terms]) OR (H7N9 subtype)) OR (H7N9 subtypes)) OR (H7N9 Virus)) OR (H7N9 Viruses)) OR (Virus H7N9)) OR (H7N9)** Filters: **Case Reports, Clinical Study, Clinical Trial, Randomized Controlled Trial, Chinese, English, from 2013/3/1 - 2024/3/31**<br>("influenza a virus, h7n9 subtype"[MeSH Terms] OR ("influenza a virus, h7n9 subtype"[MeSH Terms] OR "h7n9 subtype influenza a virus"[All Fields] OR ("h7n9"[All Fields] AND "subtype"[All Fields]) OR "h7n9 subtype"[All Fields]) OR ("influenza a virus, h7n9 subtype"[MeSH Terms] OR "h7n9 subtype influenza a virus"[All Fields] OR ("h7n9"[All Fields] AND "subtypes"[All Fields]) OR "h7n9 subtypes"[All Fields]) OR ("influenza a virus, h7n9 subtype"[MeSH Terms] OR "h7n9 subtype influenza a virus"[All Fields] OR ("h7n9"[All Fields] AND "virus"[All Fields]) OR "h7n9 virus"[All Fields]) OR ("influenza a virus, h7n9 subtype"[MeSH Terms] OR "h7n9 subtype influenza a virus"[All Fields] OR ("h7n9"[All Fields] AND "viruses"[All Fields]) OR "h7n9 viruses"[All Fields]) OR ("influenza a virus, h7n9 subtype"[MeSH Terms] OR "h7n9 subtype influenza a virus"[All Fields] OR ("virus"[All Fields] AND "h7n9"[All Fields]) OR "virus h7n9"[All Fields]) OR ("influenza in birds"[MeSH Terms] OR ("influenza"[All Fields] AND "birds"[All Fields]) OR "influenza in birds"[All Fields] OR "h7n9"[All Fields])) AND ((casereports[Filter] OR clinicalstudy[Filter] OR clinicaltrial[Filter] OR randomizedcontrolledtrial[Filter]) AND (2013/3/1:2024/3/31[pdat]) AND (chinese[Filter] OR english[Filter]))	225	02:17:11

Supplementary Figure S2： Search strategy for EMBASE database and result

History Save | Delete | Print view | Export | Email Combine using And Or Collapse

☐	**#5**	#2 AND (**'case report'**/de OR **'clinical article'**/de OR **'randomized controlled trial'**/de OR **'retrospective study'**/de) AND [01-03-2013]/sd NOT [01-04-2024]/sd AND ([chinese]/lim OR [english]/lim)	371
☐	**#4**	#2 AND (**'case report'**/de OR **'clinical article'**/de OR **'randomized controlled trial'**/de OR **'retrospective study'**/de) AND [01-03-2013]/sd NOT [01-04-2024]/sd	371
☐	**#3**	#2 AND (**'case report'**/de OR **'clinical article'**/de OR **'randomized controlled trial'**/de OR **'retrospective study'**/de)	374
☐	**#2**	**'h7n9 subtype'**:ab,ti OR **'h7n9 subtypes'**:ab,ti OR **'h7n9 virus'**:ab,ti OR **'h7n9 viruses'**:ab,ti OR **'virus h7n9'**:ab,ti OR **'avian influenza a h7n9'**:ab,ti OR **'h7n9'**	2,965
☐	**#1**	**'influenza a virus (h7n9)'**/exp	2,181

Supplementary Figure S3: .Search strategy for Web of science database and result

4/5 Combine Sets Export Clear History

Set	Query	Results
5	((((((((TS=(Influenza A Virus H7N9 Subtype)) OR TS=(H7N9 subtype)) OR TS=(H7N9 subtypes)) OR TS=(H7N9 Virus)) OR TS=(H7N9 Viruses)) OR TS=(Virus H7N9)) OR TS=(avian influenza A H7N9)) OR TS=(H7N9)) AND ALL=(case reports OR case report OR retrospective studies OR case series))	321
4	(((((((TS=(Influenza A Virus H7N9 Subtype)) OR TS=(H7N9 subtype)) OR TS=(H7N9 subtypes)) OR TS=(H7N9 Virus)) OR TS=(H7N9 Viruses)) OR TS=(Virus H7N9)) OR TS=(avian influenza A H7N9)) OR TS=(H7N9) and Article or Letter or Editorial Material (Document Types) and English or Chinese (Languages)	2,276
3	(((((((TS=(Influenza A Virus H7N9 Subtype)) OR TS=(H7N9 subtype)) OR TS=(H7N9 subtypes)) OR TS=(H7N9 Virus)) OR TS=(H7N9 Viruses)) OR TS=(Virus H7N9)) OR TS=(avian influenza A H7N9)) OR TS=(H7N9) and Article or Letter or Editorial Material (Document Types)	2,285
2	(((((((TS=(Influenza A Virus H7N9 Subtype)) OR TS=(H7N9 subtype)) OR TS=(H7N9 subtypes)) OR TS=(H7N9 Virus)) OR TS=(H7N9 Viruses)) OR TS=(Virus H7N9)) OR TS=(avian influenza A H7N9)) OR TS=(H7N9)	2,633

Supplementary Figure S4: Search strategy for CNKI database and result

检索条件	检索范围	检索时间
(主题：H7N9 + h7n9禽流感 + h7n9流感 + h7n9病毒 + h7n9亚型) AND (篇名：病例 + 例 + 病例报告 + 病例对照 + 病例报道(精确)) AND (篇名：人(精确))	资源范围：学术期刊; 中英文扩展; 时间范围：出版年度：2013 到 2024,更新时间：不限; 来源类别：全部期刊;	2024-06-10 22:06:49

共找到 309 条结果

Supplementary Figure S5: Search strategy for VIP database and result

编号	检索结果	检索表达式
1#	318	((题名或关键词=H7N9 AND ((((题名=病例 OR 题名=例) OR 题名=病例报告) OR 题名=病例报道) OR 题名=病例对照)) AND 题名=人) AND (years:[2013 TO 2024]) AND (学科:医药卫生)

Supplementary Figure S6: Search strategy for Wangfang database and result

删除已选

全选	文献类型	检索式	检索结果	检索时间	操作
1	期刊	(主题:("H7N9") and 题名或关键词:(病例 or 病例报告 or 病例报道 or 病例资料 or 病例分析 or 病例讨论 or 随机对照) and 题名或关键词:(人)) and 出版时间:[2013-03-01 TO 2024-03-31)	227	2024-06-10	删除

Table S1 PRISMA 2020 Checklist

Section and Topic	Item #	Checklist item	Location where item is reported
**TITLE**			
Title	1	Identify the report as a systematic review.	Page 1
**ABSTRACT**			
Abstract	2	See the PRISMA 2020 for Abstracts checklist.	Page 2
**INTRODUCTION**			
Rationale	3	Describe the rationale for the review in the context of existing knowledge.	Page 3
Objectives	4	Provide an explicit statement of the objective(s) or question(s) the review addresses.	Page 3
**METHODS**			
Eligibility criteria	5	Specify the inclusion and exclusion criteria for the review and how studies were grouped for the syntheses.	Page 4
Information sources	6	Specify all databases, registers, websites, organisations, reference lists and other sources searched or consulted to identify studies. Specify the date when each source was last searched or consulted.	Page 4
Search strategy	7	Present the full search strategies for all databases, registers and websites, including any filters and limits used.	Page 4<br>Figure S1 -S6,TableS2
Selection process	8	Specify the methods used to decide whether a study met the inclusion criteria of the review, including how many reviewers screened each record and each report retrieved, whether they worked independently, and if applicable, details of automation tools used in the process.	Page 4
Data collection process	9	Specify the methods used to collect data from reports, including how many reviewers collected data from each report, whether they worked independently, any processes for obtaining or confirming data from study investigators, and if applicable, details of automation tools used in the process.	Page 4
Data items	10a	List and define all outcomes for which data were sought. Specify whether all results that were compatible with each outcome domain in each study were sought (e.g. for all measures, time points, analyses), and if not, the methods used to decide which results to collect.	Page 5
	10b	List and define all other variables for which data were sought (e.g. participant and intervention characteristics, funding sources). Describe any assumptions made about any missing or unclear information.	Page 5
Study risk of bias assessment	11	Specify the methods used to assess risk of bias in the included studies, including details of the tool(s) used, how many reviewers assessed each study and whether they worked independently, and if applicable, details of automation tools used in the process.	Page 5
Effect measures	12	Specify for each outcome the effect measure(s) (e.g. risk ratio, mean difference) used in the synthesis or presentation of results.	Page 5
Synthesis methods	13a	Describe the processes used to decide which studies were eligible for each synthesis (e.g. tabulating the study intervention characteristics and comparing against the planned groups for each synthesis (item #5)).	Page 5
	13b	Describe any methods required to prepare the data for presentation or synthesis, such as handling of missing summary statistics, or data conversions.	Page 5
	13c	Describe any methods used to tabulate or visually display results of individual studies and syntheses.	Table 1,TableS3
	13d	Describe any methods used to synthesize results and provide a rationale for the choice(s). If meta-analysis was performed, describe the model(s), method(s) to identify the presence and extent of statistical heterogeneity, and software package(s) used.	Page 5
	13e	Describe any methods used to explore possible causes of heterogeneity among study results (e.g. subgroup analysis, meta-regression).	NA
	13f	Describe any sensitivity analyses conducted to assess robustness of the synthesized results.	NA
Reporting bias assessment	14	Describe any methods used to assess risk of bias due to missing results in a synthesis (arising from reporting biases).	NA
Certainty	15	Describe any methods used to assess certainty (or confidence) in the body of evidence for an outcome.	NA

Table S1 PRISMA 2020 Checklist

Section and Topic	Item #	Checklist item	Location where item is reported
assessment			
**RESULTS**			
Study selection	16a	Describe the results of the search and selection process, from the number of records identified in the search to the number of studies included in the review, ideally using a flow diagram.	Page 6 Figure1
	16b	Cite studies that might appear to meet the inclusion criteria, but which were excluded, and explain why they were excluded.	NA
Study characteristics	17	Cite each included study and present its characteristics.	Table 1,TableS3
Risk of bias in studies	18	Present assessments of risk of bias for each included study.	Table 1,TableS3
Results of individual studies	19	For all outcomes, present, for each study: (a) summary statistics for each group (where appropriate) and (b) an effect estimate and its precision (e.g. confidence/credible interval), ideally using structured tables or plots.	Table 1,TableS3
Results of syntheses	20a	For each synthesis, briefly summarise the characteristics and risk of bias among contributing studies.	Table 1,TableS3
	20b	Present results of all statistical syntheses conducted. If meta-analysis was done, present for each the summary estimate and its precision (e.g. confidence/credible interval) and measures of statistical heterogeneity. If comparing groups, describe the direction of the effect.	Table 1,Table 2
	20c	Present results of all investigations of possible causes of heterogeneity among study results.	NA
	20d	Present results of all sensitivity analyses conducted to assess the robustness of the synthesized results.	NA
Reporting biases	21	Present assessments of risk of bias due to missing results (arising from reporting biases) for each synthesis assessed.	NA
Certainty of evidence	22	Present assessments of certainty (or confidence) in the body of evidence for each outcome assessed.	NA
**DISCUSSION**			
Discussion	23a	Provide a general interpretation of the results in the context of other evidence.	Page 9
	23b	Discuss any limitations of the evidence included in the review.	Page 10
	23c	Discuss any limitations of the review processes used.	Page 10
	23d	Discuss implications of the results for practice, policy, and future research.	Page 9-11
**OTHER INFORMATION**			
Registration and protocol	24a	Provide registration information for the review, including register name and registration number, or state that the review was not registered.	Page 4
	24b	Indicate where the review protocol can be accessed, or state that a protocol was not prepared.	Page 4
	24c	Describe and explain any amendments to information provided at registration or in the protocol.	NA
Support	25	Describe sources of financial or non-financial support for the review, and the role of the funders or sponsors in the review.	Page 12
Competing interests	26	Declare any competing interests of review authors.	Page 12
Availability of data, code and other materials	27	Report which of the following are publicly available and where they can be found: template data collection forms; data extracted from included studies; data used for all analyses; analytic code; any other materials used in the review.	Page 12

NA: Not applicable

Supplementary Table S2: Search databases and results.

Database	Result number
PubMed	225
EMBASE	371
Web of Science	321
CNKI	309
VIP	318
Wangfang	227
**Total**	**1771**

Table S3. Detailed information on the included articles and cases

Study	Published year	Country	Age	Gender	Underlying disease	TFIOTOT	TFDOTD	Prognosis	Quality assessment
Chen[7]	2013	China	39	Male	Yes	unused	25	Died	high
Li[[8]	2013	China	79	Male	None	—	10	Survived	Moderate
Li[8]	2013	China	67	Male	Yes	—	23	Survived	
Lv[9]	2013	China	41	Male	No	2	2	Survived	Moderate
Xia[10]	2013	China	42	Male	None	7	7	Survived	high
Xie[11]	2013	China	38	Male	Yes	unused	19	Died	Moderate
Liu[12]	2013	China	36	Male	None	0	0	Survived	Low
Shen[13]	2013	China	54	Female	None	4	4	Survived	Low
Gao[1]	2013	China	27	Male	Yes	12	31	Died	Low
Gao[1]	2013	China	87	Male	Yes	13	40	Died	
Gao[1]	2013	China	35	Female	Yes	14	17	Died	
Lu[14]	2013	China	65	Male	Yes	3	—	Survived	Moderate
Lu[14]	2013	China	73	Male	Yes	6	—	Died	
Lu[14]	2013	China	67	Male	No	8	—	Survived	
Qi[15]	2013	China	32	Female	No	3	10	Died	Moderate
Qi[15]	2013	China	60	Male	Yes	11	23	Died	
Zhu[16]	2013	China	15	Male	No	1	1	Survived	Low
Mei[17]	2013	China	3.5	Male	No	0	1	Survived	Low
Feng[18]	2013	China	77	Male	Yes	8	10	Died	High
Liu[19]	2013	China	65	Male	Yes	2	2	Survived	Moderate
Shou[20]	2013	China	48	Male	None	8	8	Survived	Low
Shou[20]	2013	China	69	Male	No	8	8	Died	
Song[21]	2013	China	55	Male	Yes	6	9	Survived	High
Tang[22]	2013	China	65	Male	None	2	5	Survived	Low
Zhou[23]	2013	China	22	Female	None	unused	3	Survived	Low
Zhou[23]	2013	China	26	Female	None	unused	0	Survived	
Qiao[24]	2014	China	87	Male	Yes	6	14	Died	Low
Nicholls[25]	2014	Hongkong	65	Male	Yes	unused	6	Died	Low
Ho[26]	2014	Hongkong	36	Female	No	8	9	Survived	Moderate
Song[27]	2014	China	7	Female	None	1	1	Survived	Moderate
Jin[28]	2014	China	42	Male	Yes	6	6	Survived	Moderate
Jin[28]	2014	China	37	Male	No	19	19	Survived	
Qi[29]	2014	China	25	Female	No	10	10	Survived	Moderate
Wang[30]	2014	China	64	Male	Yes	7	7	Died	Moderate
Wang[30]	2014	China	54	Male	No	8	8	Died	
Wang[30]	2014	China	67	Female	Yes	14	14	Died	
Wang[31]	2014	China	38	Male	None	4	8	Survived	High
Chen[32]	2014	China	5	Male	None	1	3	Survived	Low

Chen[32]	2014	China	4	Female	None	5	8	Survived	
Chen[33]	2014	China	68	Male	Yes	4	—	Died	Moderate
Ding[34]	2014	China	56	Male	Yes	—	4	Died	Low
Fu[35]	2014	China	60	Male	None	—	6	Survived	Moderate
Guo[36]	2014	China	38	Male	None	unused	30	Died	Moderate
Hu[37]	2014	China	52	Female	None	35	36	Died	Low
Hu[37]	2014	China	56	Male	None	—	10	Died	
Huang[38]	2014	China	62	Male	None	6	6	Survived	Moderate
Xie[39]	2014	China	61	Female	No	—	9	Died	Moderate
Li[40]	2014	China	64	Male	Yes	—	8	Survived	Low
Lin[41]	2014	China	71	Male	None	6	9	Survived	Moderate
Liu[42]	2014	China	74	Male	None	4	—	Died	Low
Liu[43]	2014	China	54	Male	None	10	11	Died	Moderate
Luo[44]	2014	China	65	Male	None	5	6	Survived	Moderate
Lv[45]	2014	China	76	Male	Yes	—	12	Died	Moderate
Shao[46]	2014	China	69	Male	Yes	8	8	Died	Moderate
Sun[47]	2014	China	14	Female	No	—	2	Survived	Low
Yang[48]	2014	China	69	Male	None	5	5	Survived	Moderate
Ye[49]	2014	China	4.9	Female	None	0	1	Survived	Low
Ye[49]	2014	China	29	Male	None	5	7	Survived	
Zhang[50]	2014	China	38	Male	None	—	9	Survived	Low
Zhu[51]	2014	China	69	Female	None	3	3	Survived	Low
Zhu[51]	2014	China	31	Female	None	6	6	Survived	
Zhu[51]	2014	China	76	Female	None	7	7	Survived	
Zhu[52]	2014	China	50	Female	None	6	6	Survived	Moderate
Zhuang[53]	2014	China	38	Female	Yes	7	8	Survived	High
Wang[54]	2015	China	38	Male	No	unused	8	Died	Moderate
Cui[55]	2015	China	37	Female	No	6	6	Survived	Moderate
Cui[55]	2015	China	33	Female	No	8	8	Survived	
Dai[56]	2015	China	58	Female	Yes	—	6	Died	Low
Dai[56]	2015	China	61	Female	Yes	—	7	Died	
Wang[57]	2015	China	29	Female	Yes	6	6	Died	High
Pan[58]	2015	China	31	Male	Yes	6	8	Died	High
William[59]	2015	Malaysia	66	Female	No	6	10	Survived	High
Hu[60]	2015	China	61	Female	None	11	11	Died	Low
Feng[61]	2015	China	87	Male	No	5	5	Died	Low
Feng[61]	2015	China	88	Male	Yes	5	5	Died	
Zhang[62]	2015	China	86	Male	Yes	6	6	Died	Moderate
Zhang[62]	2015	China	58	Male	Yes	14	14	Died	
Chen[63]	2015	China	57	Female	Yes	5	9	Survived	Low
Chen[63]	2015	China	43	Female	None	6	9	Survived	
Chen[63]	2015	China	80	Male	Yes	—	0	Died	
Chen[63]	2015	China	78	Male	Yes	—	3	Died	
Chen[63]	2015	China	34	Female	None	—	11	Survived	

Guo[64]	2015	China	71	Male	Yes	7	11	Survived	Moderate
Kong[65]	2015	China	31	Male	No	6	7	Survived	High
Liao[66]	2015	China	75	Female	Yes	5	11	Survived	Moderate
Ma[67]	2015	China	77	Male	Yes	—	5	Died	Low
Liu[68]	2015	China	23	Male	None	1	1	Survived	Moderate
Liu[68]	2015	China	21	Female	None	—	7	Died	
Shen[69]	2015	China	53	Male	None	5	6	Survived	Low
Shen[69]	2015	China	73	Male	Yes	5	5	Survived	
Su[70]	2015	China	51	Male	None	4	11	Survived	Low
Su[70]	2015	China	52	Male	None	4	5	Survived	
Su[70]	2015	China	82	Male	None	6	11	Survived	
Su[70]	2015	China	42	Male	None	9	11	Survived	
Wang[71]	2015	China	21	Female	No	—	5	Survived	Low
Wang[71]	2015	China	60	Male	Yes	—	5	Died	
Wang[[72]	2015	China	65	Male	Yes	—	9	Died	Low
Wang[73]	2015	China	4	Male	None	0	0	Survived	Low
Wang[73]	2015	China	36	Male	No	5	5	Survived	
Wu[74]	2015	China	1.9	Female	None	—	2	Survived	Low
Zhang[75]	2015	China	3	Male	None	2	2	Survived	Low
Zhao[76]	2015	China	35	Male	None	—	6	Survived	Low
Chen[77]	2016	China	57	Male	Yes	9	10	Died	Moderate
Chen[77]	2016	China	71	Male	Yes	21	22	Died	
Qian[78]	2016	China	55	Male	Yes	—	10	Survived	Moderate
Liu[79]	2016	China	66	Female	None	—	21	Died	Low
Hu[80]	2016	China	61	Female	No	6	5	Died	Moderate
Gao[81]	2016	China	44	Female	None	—	7	Died	Low
Jiang[82]	2016	China	39	Female	No	8	10	Died	Low
Wang[83]	2016	China	50	Male	Yes	5	5	Survived	Moderate
Weng[84]	2016	China	55	Male	None	4	5	Survived	Low
Weng[84]	2016	China	79	Male	None	6	7	Died	
Xu[85]	2016	China	63	Male	Yes	10	14	Died	High
Yi[86]	2016	China	57	Male	Yes	1	1	Died	Low
Yin[87]	2016	China	49	Male	No	4	4	Survived	Moderate
Zhang[88]	2016	China	35	Male	None	—	1	Survived	Low
Ke[89]	2017	China	56	Male	Yes	3	6	Died	Low
Zhang[90]	2017	China	43	Female	None	1	—	Survived	Moderate
Zhang[90]	2017	China	62	Female	Yes	9	—	Survived	
Nie[91]	2017	China	44	Female	None	18	18	Survived	Moderate
Feng[92]	2017	China	24	Female	No	4	4	Died	Moderate
Zhang[93]	2017	China	62	Male	No	0	0	Survived	Low
Zhang[93]	2017	China	66	Male	Yes	5	5	Died	
Zeng[94]	2017	China	46	Female	None	—	7	Survived	Moderate
Cai[95]	2017	China	56	Male	Yes	6	6	Survived	Moderate
Chen[96]	2017	China	76	Male	Yes	5	6	Died	Moderate

Gong[97]	2017	China	57	Male	Yes	9	9	Survived	Moderate
Hu[98]	2017	China	29	Male	No	4	8	Survived	Moderate
Hu[98]	2017	China	56	Female	Yes	6	21	Survived	
Jiao[99]	2017	China	62	Male	None	13	13	Died	Moderate
Li[100]	2017	China	36	Male	None	—	5	Survived	Low
Liu[101]	2017	China	41	Female	No	7	7	Died	Moderate
Liu[102]	2017	China	52	Male	None	6	8	Survived	Low
Shuai[103]	2017	China	38	Male	Yes	—	4	Died	Low
Wang[104]	2017	China	48	Male	None	10	10	Survived	Low
Wang[105]	2017	China	50	Male	None	11	12	Survived	Moderate
Wang[106]	2017	China	63	Male	Yes	7	—	Survived	
Wang[106]	2017	China	68	Male	Yes	7	—	Survived	Low
Wang[106]	2017	China	68	Male	Yes	7	—	Survived	
Wu[107]	2017	China	53	Male	None	6	4	Died	Low
Zhang[108]	2017	China	56	Female	None	7	9	Survived	Low
Zhang[109]	2017	China	64	Female	None	—	6	Survived	Low
Zhao[110]	2017	China	60	Male	Yes	0	1	Died	Low
Zhao[110]	2017	China	54	Male	Yes	—	6	Died	
Zhong[111]	2017	China	38	Male	None	—	6	Survived	Low
Luo[112]	2018	China	80	Female	Yes	8	9	Survived	High
Wang[113]	2018	China	39	Female	No	6	6	Survived	Moderate
Wang[113]	2018	China	66	Male	Yes	9	12	Died	
Wang[114]	2018	China	28	Female	Yes	2	7	Survived	Moderate
Guo[115]	2018	China	3	Female	None	unused	7	Died	High
Cao[116]	2018	China	60	Male	Yes	—	3	Survived	
Cao[116]	2018	China	75	Male	Yes	—	8	Died	Low
Cao[116]	2018	China	48	Female	Yes	—	10	Survived	
Dai[117]	2018	China	53	Male	None	0	6	Died	Moderate
Geng[118]	2018	China	65	Female	Yes	4	7	Survived	Moderate
Gong[119]	2018	China	47	Male	Yes	—	8	Survived	Low
Gu[120]	2018	China	38	Male	Yes	6	6	Died	Moderate
Guo[121]	2018	China	51	Male	No	5	5	Survived	High
Guo[122]	2018	China	49	Male	No	4	4	Died	Moderate
He[123]	2018	China	40	Male	None	8	8	Survived	Low
He[123]	2018	China	61	Male	None	11	13	Survived	
Ji[124]	2018	China	34	Male	No	—	6	Died	Low
Jiang[125]	2018	China	75	Male	None	8	8	Died	Moderate
Jiang[126]	2018	China	52	Male	Yes	5	7	Died	
Jiang[126]	2018	China	58	Female	Yes	7	12	Survived	Low
Jiang[126]	2018	China	56	Male	Yes	9	11	Died	
Li[127]	2018	China	82	Male	None	3	7	Survived	Moderate
Li[128]	2018	China	67	Male	None	2	6	Survived	High
Liao[129]	2018	China	51	Male	Yes	—	2	Died	Low
Liu[130]	2018	China	63	Female	None	4	11	Survived	Moderate

Liu[131]	2018	China	63	Male	No	4	5	Survived	Low
Liu[131]	2018	China	62	Male	Yes	—	11	Died	
Liu[132]	2018	China	66	Female	None	15	15	Died	Low
Liu[133]	2018	China	61	Female	Yes	—	9	Died	Moderate
Luo[134]	2018	China	43	Female	No	—	3	Survived	Low
Luo[134]	2018	China	34	Female	Yes	—	7	Survived	
Luo[134]	2018	China	55	Male	No	—	9	Survived	
Luo[134]	2018	China	4.5	Female	No	—	10	Survived	
Luo[134]	2018	China	28	Male	No	—	16	Survived	
Ma[135]	2018	China	39	Female	No	13	13	Survived	Moderate
Shi[136]	2018	China	56	Male	Yes	14	15	Survived	Moderate
Wang[137]	2018	China	36	Female	None	6	6	Survived	Low
Wang[137]	2018	China	36	Female	None	10	10	Survived	
Wu[138]	2018	China	52	Male	Yes	—	3	Died	Low
Wu[138]	2018	China	38	Male	Yes	—	7	Died	
Xu[139]	2018	China	33	Male	Yes	2	7	Survived	Low
Xu[139]	2018	China	61	Male	No	—	15	Died	
Xue[[140]	2018	China	74	Male	Yes	—	10	Survived	Low
Xue[140]	2018	China	73	Male	Yes	—	11	Died	
Ying[141]	2018	China	71	Male	No	4	4	Died	Low
Yu[142]	2018	China	67	Male	No	6	9	Died	Moderate
Tashi[143]	2018	China	40	Male	Yes	—	2	Survived	Low
Tashi[143]	2018	China	41	Male	None	—	6	Survived	
Tashi[143]	2018	China	43	Male	None	—	6	Survived	
Zhang[144]	2018	China	35	Female	None	5	5	Survived	Moderate
Zhang[145]	2018	China	67	Male	Yes	—	7	Died	Low
Zhang[146]	2018	China	44	Male	None	—	6	Survived	Low
Zhong[147]	2018	China	44	Male	Yes	10	10	Survived	High
Zhou[148]	2018	China	58	Male	Yes	13	13	Died	Moderate
Zhu[149]	2018	China	22	Female	None	—	6	Died	Low
Zhu[149]	2018	China	3.3	Female	None	—	10	Died	
He[150]	2019	China	35	Male	None	5	5	Survived	High
Liu[151]	2019	China	68	Female	Yes	0	0	Survived	High
Huang[152]	2019	China	58	Male	Yes	7	7	Died	High
Huang[153]	2019	China	28	Male	None	3	3	Died	High
Zhang[154]	2019	China	59	Male	None	0	1	Survived	Moderate
Zhang[154]	2019	China	62	Female	None	1	1	Died	
Zhang[154]	2019	China	30	Male	None	10	10	Survived	
Chen[155]	2019	China	34	Male	No	—	4	Survived	Moderate
Luo[156]	2019	China	62	Female	No	—	7	Survived	Low
Luo[156]	2019	China	44	Male	Yes	—	8	Survived	
Luo[156]	2019	China	51	Male	No	—	8	Survived	
Luo[156]	2019	China	54	Female	Yes	—	8	Survived	
Luo[156]	2019	China	61	Male	No	—	10	Died	Low

Luo[[156]	2019	China	52	Male	No	—	5	Survived	
Qi[157]	2019	China	66	Male	None	—	12	Died	Low
Qi[157]	2019	China	61	Female	None	—	16	Died	
Tao[158]	2019	China	57	Female	Yes	7	7	Survived	Moderate
Wei[159]	2019	China	48	Male	No	10	9	Survived	Moderate
Wei[159]	2019	China	79	Male	Yes	—	4	Died	
Xia[160]	2019	China	70	Female	Yes	3	8	Died	Low
Xia[160]	2019	China	52	Male	No	4	5	Survived	
Xia[160]	2019	China	70	Male	Yes	5	7	Survived	
Xia[160]	2019	China	73	Male	Yes	7	8	Died	
Xia[160]	2019	China	60	Male	No	9	12	Died	
Zhao[161]	2019	China	54	Male	Yes	7	7	Survived	Moderate
Zhang[162]	2019	China	64	Female	Yes	—	13	Died	Low
Zhang[162]	2019	China	74	Male	None	—	6	Survived	
Zhou[163]	2019	China	43	Female	None	9	7	Survived	Moderate
Zhou[163]	2019	China	23	Male	None	16	14	Survived	
Huang[164]	2020	China	82	Male	Yes	11	11	Died	Moderate
Li[165]	2020	China	54	Male	None	7	7	Survived	Moderate
Li[165]	2020	China	45	Female	None	10	15	Died	
Lu[166]	2020	China	37	Male	Yes	—	13	Died	Moderate
Pan[167]	2020	China	82	Male	Yes	—	10	Died	Low
Wang[168]	2020	China	57	Male	None	—	15	Survived	Low
Wang[169]	2021	China	46	Female	None	4	4	Survived	Moderate

None: Non-avaible; **Yes**: There are underlying diseases; **NO**: No underlying disease

TFIOTOT: Time from illness onset to Oseltamivir therapy

TFDOTD: Time from disease onset to diagnosis

Reference numbers correspond to those in the main manuscript.
